# Evolution and comparative transcriptome analysis of glucosinolate pathway genes in *Brassica napus* L.

**DOI:** 10.3389/fpls.2024.1483635

**Published:** 2024-12-10

**Authors:** Shiying Liu, Zexuan Wu, Xingying Chen, Zhuo Chen, Yibing Shen, Salman Qadir, Huafang Wan, Huiyan Zhao, Nengwen Yin, Jiana Li, Cunmin Qu, Hai Du

**Affiliations:** ^1^ College of Agronomy and Biotechnology, Chongqing Engineering Research Center for Rapeseed, Southwest University, Chongqing, China; ^2^ Academy of Agricultural Sciences, Southwest University, Chongqing, China

**Keywords:** glucosinolate, evolution, *Brassica napus*, pathway, transcriptome

## Abstract

Glucosinolates (GSLs) are important secondary metabolites abundantly distributed in Brassicaceae plants, whose degradation products benefit plant resistance but are regarded as disadvantageous factors for human health. Thus, reducing GSL content is an important goal in the breeding program in crops, such as *Brassica napus*. In this study, 1280 genes in the GSL pathway were identified from 14 land plant genomes, which are specifically distributed in Brassicaceae and are extensively expanded in *B. napus*. Most GSL pathway genes had many positive selection sites, especially the encoding genes of transcription factors (TFs) and structural genes involved in the GSL breakdown process. There are 344 genes in the GSL pathway in the *B. napus* genome, which are unequally distributed on the 19 chromosomes. Whole-genome duplication mainly contributed to the gene expansion of the GSL pathway in *B. napus*. The genes in GSL biosynthesis were regulated by various TFs and *cis*-elements in *B. napus* and mainly response to abiotic stress and hormone induction. A comparative transcriptome atlas of the roots, stems, leaves, flowers, siliques, and seeds of a high- (ZY821), and a low-GSL-content (ZS11) cultivar was constructed. The features of the two cultivars may be attributed to diverse expression differences in each organ at different stages, especially in seeds. In all, 65 differential expressed genes (DEGs) concentrated on the core structure pathway were inferred to mainly influence the GSL contents between ZY821 and ZS11. This study provides an important RNA-seq dataset and diverse gene resources for future manipulating GSLs biosynthesis and distribution in *B. napus* using molecular breeding methods.

## 1 Introduction

Glucosinolates (GSLs) are sulfur- and nitrogen-containing secondary metabolites with a variable side chain widely present in Brassicales, especially Brassicaceae plants, such as *Arabidopsis thaliana*, *Brassica napus*, *B. rapa* and *B. oleracea* (Fahey et al., 2001; Yan and Chen, 2007). GSLs are hydrolyzed by endogenous thioglucosidases and myrosinases, producing a variety of breakdown products such as isothiocyanates, nitriles, and oxazolidine-2-thione. These products are involved in many biological and economic processes, including avoiding herbivores, pathogen defense, and determining flavor, smell, and anti-carcinogenic properties (Hopkins et al., 2009; Dinkova-Kostova and Kostov, 2012; Santolamazza-Carbone et al., 2013).

To date, more than 200 kinds of GSLs have been identified in plants. Based on the amino acid precursors, GSLs are classified into three major groups: aliphatic GSL (AGSL) derived from methionine (Met) or other aliphatic amino acids, indole GSL (IGSL) derived from tryptophan (Trp), and aromatic GSL derived from phenylalanine (Phe) or tyrosine (Tyr) (Fahey et al., 2001; Sonderby et al., 2010). GSL biosynthesis and hydrolysis require the involvement of multiple enzymes and regulatory factors. Although very little is known about the genes in GSLs-containing crops, most of the structural and regulating genes have been characterized in model plant *Arabidopsis*, forming a systematic GSL pathway (Sonderby et al., 2010). Among the three major GSL types, the genes involved in AGSL and IGSL pathways have been well investigated, but those in aromatic GSL are less known yet. The GSL biosynthesis pathway is comprised of three stages: side-chain elongation, core structure biosynthesis, and subsequent secondary modification, with the core structure biosynthesis process is shared by AGSL and IGSL pathways while the other two are different (Halkier and Gershenzon, 2006; Burow et al., 2010; Mostafa et al., 2016). For example, in the AGSL pathway, the side-chain elongation process mainly consists of four stages catalyzed by different enzymes. These stages include deamination caused by the branched-chain amino acid aminotransferase 4 (BCAT4) (Schuster et al., 2006), condensation with acetyl-CoA, isomerization induced by Leus, and oxidative decarboxylation activated by isopropylmalate dehydrogenases (IPMDHs) (Field et al., 2004). In contrast, in the IGSL pathway, the amino acid precursors can directly form the initiator (aldoxime) of the core structure biosynthesis through catalysis by the CYP79 subfamily. Besides the structural genes, some regulatory genes involved in the GSL pathway have been proved to regulate the expression of structural genes, including bHLH, Dof, and MYB genes, etc (Frerigmann et al., 2014; Gigolashvili et al., 2007; Schweizer et al., 2013; Skirycz et al., 2006). For example, MYB28, MYB29, and MYB76 could specifically activate the expressions of methylthioalkylmalate synthase 3 (MAM3), CYP79F1, and CYP83A1 genes in the AGSL pathway (Gigolashvili et al., 2007; Sawada et al., 2009; Sonderby et al., 2010; Li et al., 2013).

*Brassica napus* is one of the main oil crops in the world, which provides the most source of edible vegetables and oil in China. Given some degradation products of GSL present in food tissues have toxic and anti-nutritional effects on humans and animals (Kliebenstein et al., 2005), breeding the cultivars with low GSL content had been an important goal for quality improvement in *B. napus*. Accordingly, many cultivars with significantly reduced GSL content have been developed in the past 30 years worldwide, such as Zhongshuang 11 (ZS11). Although this improved the edible safety of *B. napus* seeds for human health, it led to a decrease in plant resistance and an increase in microbial diseases and insect pests. To fully utilize the biological effect of GSLs, modulating the spatiotemporal biosynthesis and distribution of GSLs in different plant organs by molecular breeding methods is more feasible. Exploring the GSL pathway genes in Brassicaceae crops, such as *B. napus*, is fundamental to address this goal. Meanwhile, though many studies have proved that GSLs are present exclusively in the Brassicaceae family and even Brassicales, the evolution of this defense system in the plant kingdom at the genome-wide level is still lacking.

In this study, we first identified GSL pathway genes in the major sequenced plant genomes to investigate the evolutionary history of this pathway in plants. Based on the results that homologs were not found in most plant genomes, we further focus on 14 representative plants, including 13 dicot species and one monocot species (*Oryza sativa*), and the selection pressure of GSL pathway genes of 14 land plants was calculated subsequently. We conducted global analyses of the GSL pathway genes in the *B. napus* genome at the genome-wide level, accompanied by a series of bioinformatics assays of the candidates, including chromosomal location, collinearity relationship, gene duplication, regulatory mechanism prediction, etc. Moreover, we constructed an RNA-seq dataset of a high- (ZY821) and a low-GSL-content (ZS11) *B. napus* cultivar that includes 36 tissue/organ samples across different developmental stages. Based on the transcriptome dataset, we compared the expression profiles of GSL pathway genes in different organs between these two cultivars, and the significantly differentially expressed genes were screened. This study will facilitate gene functional research and molecular breeding in *B. napus* toward regulatory GSL contents.

## 2 Material and methods

### 2.1 Plant materials

The high-GSL content *Brassica napus* Zhongyou 821 (ZY821) pure seeds (GSL=152.215 μmol/g) and the low-GSL content *Brassica napus* Zhongshuang 11 (ZS11) pure seeds (GSL=13.913 μmol/g) were obtained from the College of Agriculture and Biotechnology, Southwest University (Beibei, Chongqing, China). The seeds were grown in the field at Southwest University (Chongqing, China). Thirty-six different tissues were collected from the two cultivars, including roots, stems, leaves and flowers at the seedling (S-Ro, S-St, S-Le), bud (B-Ro, B-St, B-Le), and full-bloom stages (F-Ro, F-St, F-Le, F-Fl), respectively; silique (Si) at 3 and 7 days after flowering (DAF); and seeds (Se) at 14, 21, 28, 35, 42, and 49 DAF at the seed maturation stage. Each sample contained the tissues from at least three plants. All samples were taken simultaneously at different developmental periods, and immediately frozen in liquid nitrogen, and then were stored at -80° for RNA extraction.

### 2.2 RNA sequencing and data analysis

EASYspin Total RNA Extraction Kit (BioMed, Beijing, China) was applied for total RNA extraction. The RNA degradation and contamination were monitored on 1% agarose gel. A gel electrophoresis and a NanoPhot-ometer^®^ spectrophotometer (IMPLEN, CA, USA) (A260/280 = 1.8-2.1; A260/230 ≥ 2.0) were integrated used to examine the quality and concentration of each total RNA sample. Sequencing libraries were generated using NEBNext Ultra™ RNA Library Prep Kit for Illumina (NEB, California, USA) following the manufacturer’s recommendations and dual index codes were added to attribute sequences to each sample. Briefly, mRNA was purified from total RNA using poly-T oligo-attached magnetic beads. Fragmentation was carried out using divalent cations under elevated temperature in NEBNext First Strand Synthesis Reaction Buffer. First strand cDNA was synthesized using random hexamer primer and M-MuLV Reverse Transcriptase. Second strand cDNA synthesis was subsequently performed using DNA Polymerase I and RNase H. Remaining overhangs was converted into blunt ends via exonuclease/polymerase activities. After adenylation of 3’ ends of DNA fragments, NEBNext Adaptor with hairpin loop structure was ligated to prepare for hybridization. To select cDNA fragments of preferentially 240 bp in length, the library fragments were purified with the AMPure XP system (Beckman Coulter, Massachusetts, USA). Then 3 μl USER Enzyme (NEB, USA) was used with size-selected, adaptor-ligated cDNA at 37°C for 15 min followed by 5 min at 95°C before PCR. Then PCR was performed with Phusion High-Fidelity DNA polymerase, Universal PCR primers, and dual indexing primers. At last, PCR products were purified with AMPure XP system and library quality was assessed on the Agilent Bioanalyzer 2100 system. The clustering of the index-coded samples was performed on a cBot Cluster Generation System using TruSeq PE Cluster Kit v4-cBot-HS (Illumia) according to the manufacturer’s instructions. After cluster generation, the libraries were sequenced on an Illumina platform, and 150 bp paired-end reads were generated. A total of 72 samples were sequenced on an Illumina Hiseq™ 2000 platform at BioMarker (Beijing, China). Raw reads were firstly processed through in-house perl scripts (PRJNA612898). In this step, clean reads were obtained by removing adapter, reads containing ploy-N and low-quality reads. At the same time, Q20, Q30, GC-content, and sequence duplication levels of the clean data were calculated. All the downstream analyses were based on clean data with high quality. Tophat2 software was used to map the high-quality clean reads with the whole-genome sequencing data of the reference genome Darmor (http://www.genoscope.cns.fr/brassicanapus/data/) for analysis and annotation.

### 2.3 Identification and analysis of glucosinolate biosynthesis genes in land plants

The query sequences of GSL pathway genes in *Arabidopsis thaliana* were sourced from TAIR (https://www.arabidopsis.org). To identify genes involved in the GSL pathway, a BLASTP search was conducted to identify the homologs using queries sequence of *Arabidopsis thaliana* from the proteome dataset of 14 plants stored in Phytozome (https://phytozome-next.jgi.doe.gov/) and GENOSCOPE (http://www.genoscope.cns.fr/brassicanapus/). The candidate genes involved in the GSL pathway were considered with an e-value of ≤ 1 × e ^-10^. To ensure the reliability of the results, candidate sequences were further validated using SMART (http://smart.emblheidelberg.de/) and PROSITE (https://prosite.expasy.org/) to confirm the presence of characteristic domains of each gene family. The phylogenetic tree of 14 land plants was constructed using the TimeTree database (Kumar et al., 2017). The candidate protein sequences from each gene family were aligned using the MAFFT online tool (https://mafft.cbrc.jp/alignment/server/), and BioEdit software respectively. Following multiple sequence alignment, a neighbor-joining (NJ) tree was constructed for each gene type (gene family) using MEGA 7.0 (Kumar et al., 2016) with the following main parameters: Poisson correction, 1000 bootstrap replicates, and pairwise deletion. The tree viewed by Figtree 1.3 software (http://tree.bio.ed.ac.uk/software/figtree/). The candidate gene was finally acquired based on a phylogeny-based classification and a homologous sequence feature.

### 2.4 Selection pressure analysis of GSL pathway genes in land plants

The CDS and protein sequences of each type of the GSL pathway homolog genes obtained in this study were aligned using MEGA7.0 respectively. Then, the aligned CDS and protein sequences of each homolog gene group were uploaded to the online website Datamonkey Adaptive Evolution Server (http://www.datamonkey.org/), and the Ka/Ks values of the homologs were calculated using the FUBAR tool (Murrell et al., 2013; Weaver et al., 2018).

### 2.5 Chromosome distribution and collinearity analysis

The information regarding chromosome length and gene locations of candidate genes were obtained from the *B. napus* genome in the GENOSCOPE database. The collinearity relationship of candidates in *B. napus*, *B. oleracea*, and *B. rapa* was analyzed by CoGe online software (https://genomevolution.org/coge/). Chromosomal localization of GSL pathway genes was mapped by MapChart software (Voorrips, 2002). The One Step MCScanX plug-in of TBtool software (Chen et al., 2020) was used to analyze and visualize the collinearity of the GSL pathway genes in *B. oleracea*, *B. rapa*, and *B. napus* genomes, and the type of the duplication event was determined based on our previous methodology (Li et al., 2020). The duplication events were accordingly defined as follows: (i)homeologous exchanges (HE): transfer of genetic information between homeologous sequences of different subgenomes; (ii) segmental exchanges (SE): transfer of genetic information between different chromosomal sequences; (iii) segmental duplications (SD): duplicated copies of chromosomal sequences. The HE, SE, and SD events were distinguished from each other based on the chromosomal homology and the colinear relationship (orthologous gene pairs in orthologous blocks) of the A_n_ (derived from *B. rapa*) and C_n_ (derived from *B. oleracea*) subgenomes and their respective progenitor genomes (*B. rapa* and *B. oleracea*) in all collinearity pairs.

### 2.6 Regulatory elements in the promoter regions of GSL pathway genes

PlantCARE database (http://bioinformatics.psb.ugent.be/webtools/plantcare/html/) (Lescot et al., 2002) was applied to predict the c*is-*acting regulatory elements in the promoter regions of candidates with default settings. The TF binding sites within the promoter sequences (-1500 bp) of candidate genes were identified using default settings in the PlantTFDB database (http://planttfdb.gao-lab.org/) (Jin et al., 2017).

### 2.7 Transcriptome analysis of ZS11 and ZY821 at different developmental stages

Gene expression levels were quantified by the cuffquant program in terms of fragments per kilobase of transcript per million fragments mapped (FPKM) (Trapnell et al., 2012). The expression values for each sample were derived from the mean expression of two biological replicates, and we defined the FPKM ≥ 1 as an expressed gene. DESeq2 was used to identify DEGs, and genes with |log2 (fold change) | ≥ 1 and p-adj (adjusted p-value) ≤ 0.01 were considered to be DEGs (Love et al., 2014). The expressed genes were used to perform the principal component analysis (PCA) based on our dataset by using the OmicShare tools (https://www.omicshare.com/tools). KEGG pathway enrichment analyses were performed using BMKCloud (www.biocloud.net). Only terms or pathways with a false discovery rate of 0.05 (p-adj ≤ 0.05) were used for the KEGG pathways analysis. The k means clustering algorithm analysis clustered the expressed genes of the two cultivars into 15 co-expression modules using the MeV v4.9 software and Java TreeView v1.1.6r4 software (Saldanha, 2004).

### 2.8 Statistical analysis

The comparisons of Ka/Ks value for different groups of genes were performed by Kruskal–Wallis ANOVA and *post hoc* Dunn’s multiple comparison test using the R package ‘multcomp’ (Hothorn et al., 2008), and P-values were adjusted with the Benjamini–Hochberg method.

## 3 Result

### 3.1 Distribution of glucosinolate metabolic pathway genes across land plants

**Figure 1:**
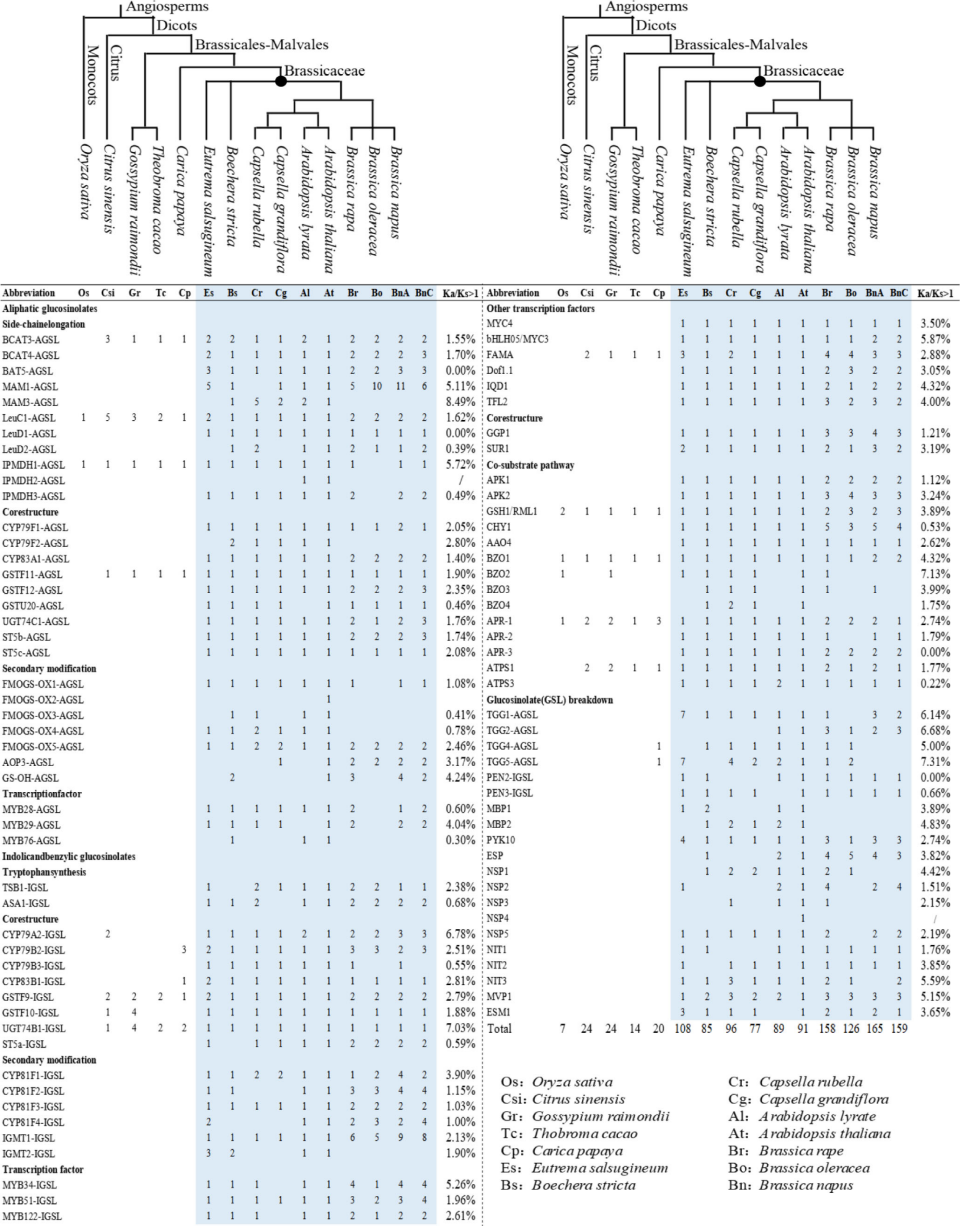
The distribution of glucosinolates (GSL) pathway genes in 14 representative land plants.

In our results, 1280 GSL pathway genes were identified from 14 representative species (**Supplementary Table S1**). They belonged to 35 gene families, with 29 gene families involved in GSLs biosynthesis pathways and six gene families in GSLs breakdown pathway, including 30 structural gene families and five TF families. In the 29 gene families, the homologs of eight gene families were specifically involved in AGSL biosynthesis (five gene families in side-chain elongation, one gene family in core structure biosynthesis, and two gene families in secondary modification), three gene families were specifically involved in IGSL biosynthesis (two gene families in side-chain elongation and one gene family in secondary modification), and 18 gene families were related to both AGSL and IGSL biosynthesis pathways (13 gene families in core structure biosynthesis and five gene families in regulatory function). Only the homologs of *isopropylmalate isomerase large subunit 1* (*LeuC1*), *glutamate-cysteine ligase 1* (*GSH1*), *benzoyloxyglucosinolate 1* (*BZO1*), and *APS reductase 1* (*APR1*) genes were found in all of the 14 representative species. However, these of *branched-chain amino acid aminotransferase 3* (*BCAT3*), *glutathione S-transferase F9* (*GSTF9*), *GSTF11*, *UDP-glucosyl transferase 74B1* (*UGT74B1*), *FAMA*, and *ATP sulfurylase 1* (*ATPS1*) gene were found in all of the dicots investigated in this study, suggesting that they were relative ancient and originated earlier (**Figure 1**). The majority of homologs belonging to the 35 gene families were only distributed in Brassicaceae, indicating the GSL gene network originated in Brassicaceae lineage (**Figure 1**). Moreover, the genes involved in secondary modification only existed in Brassicaceae, which was consistent with the fact that Brassicaceae contains a variety of GSLs with diverse side-chain structures. Furthermore, 633 of the 1280 genes (49%) belonged to *Brassica* (152 genes in *B. rapa*, 138 in *B. oleracea*, and 343 in *B. napus*), which might have resulted from the additional whole genome duplication event specific to *Brassica*.

To explore the evolutionary trends of GSL pathway genes, we performed selective pressure analysis on the protein-coding genes of the genes identified in these 14 plant species. The results showed that most of the genes involved in the GSL pathway had positive selection sites distributed throughout the gene unevenly (**Figure 1**; **Supplementary Figure S1**, **Supplementary Table S2**). The average positive selection site ratios (Ka/Ks > 1) are 9.03% for AGSL biosynthetic genes, 6.97% for IGSL biosynthetic genes, 7.48% for common pathway genes, and 10.90% for breakdown pathway genes, indicating the breakdown pathway genes underwent more positive selection pressure. The TFs (average 14.41%) exhibited a relative stronger positive selection pressure trend compared with the structural genes (average 6.66%) (Kruskal-Wallis, *P* < 0.001), indicating structural genes are relatively conserved during the evolution. Moreover, the average Ka/Ks value of TFs and breakdown pathway related genes were > 1, which is larger than that of the genes in the other processes (**Supplementary Table S2**). Furthermore, the average Ka/Ks value of the AGSL secondary modification related genes (Ka/Ks =1.08) was much higher than that of the IGSL pathway (Ka/Ks=0.544), whereas the genes in both the AGSL and IGSL side-chain elongation and core structure biosynthesis pathways were generally negatively selected (Ka/Ks < 1) (**Supplementary Table S2**). This suggested that AGSL-related genes might have played a more important role in adaptive evolution of the members of Brassicaceae.

### 3.2 Chromosomal location and collinearity of GSL pathway genes in *B. napus*

**Figure 2:**
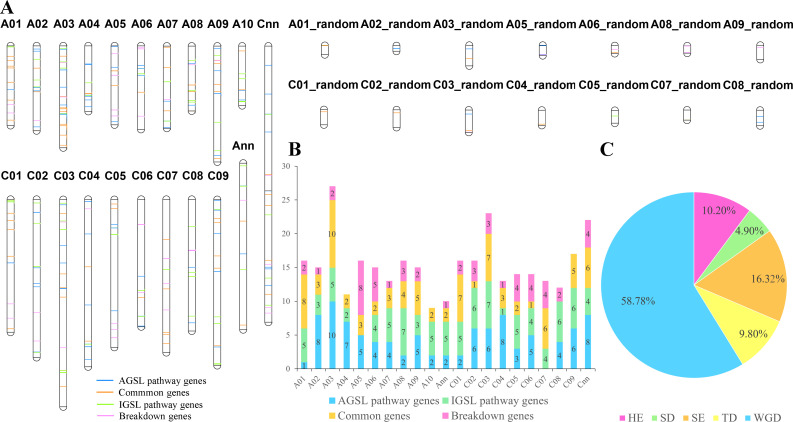
The chromosomal locations of GSL pathway genes in *B. napus*. **(A)** Chromosome positions of the 344 GSL pathway genes in *B. napus*. **(B)** The number of GSL pathway genes on each chromosome. Different colors represent the four major processes in GSL pathway. **(C)** The ratio of GSL pathway genes underwent different duplication events. HE, homologous exchange; SD, segmental duplication; SE, segmental exchange; TD, tandem duplication; WGD, whole genome duplication.

In all, 344 GSL pathway genes were distributed on all 19 chromosomes of *B. napus*, and the number of candidates in the A_n_- (170 genes) and C_n_- (174 genes) subgenomes were similar (**Figure 2A**; **Supplementary Table S3**). The distribution of AGSL and IGSL pathway genes on the two subgenomes showed a similar trend as well, but the number of genes on each chromosome was uneven (**Figure 2B**). Given that Brassicaceae species have experienced a whole-genome triplication (WGT) event compared to *A*. *thaliana*, 92 GSL pathway genes in *A. thaliana* should be expanded to 276 in *B. rapa*, 276 in *B. oleracea* and 552 genes *B. napus* genomes, respectively. However, we only found 158 (57.25%) GSL pathway genes in *B. rapa*, 126 (46.65%) GSL pathway genes in *B. oleracea*, and 324 (58.70%) GSL pathway genes in *B. napus*, indicating the occurrence of gene loss during the evolution. For instance, homologs of *CYP79B3* were only present in *B. napus* A_n_-subgenome, while homologs of *MAM3* were absent in *B. rapa*, *B. oleracea*, and *B. napus* (**Figure 2A**).

Collinearity analysis showed that 245 of the 344 genes had a collinear relationship among the homologs in *B. napus*, *B. rapa*, and/or *B. oleracea* (**Supplementary Table S4**), including 41 genes involved in side-chain elongation, 89 genes in core structure biosynthesis, 34 genes in secondary modification, 47 TFs and 34 genes in GSL breakdown process. Among the 245 genes, 144 (58.78%) were inherited from whole genome duplication (WGD) event, and 101 were derived from small-scale duplication (SSD) events, including 40 (16.32%) from SE events, 25 (10.20%) from HE events, 12 (4.90%) from SD events, and 24 (9.80%) from tandem duplication (TD) events (**Figure 2C**). These results indicated that WGD and WGT events were the main driving force for GSL pathway genes expansion in *B. napus*.

### 3.3 Regulatory relationship in the promoter regions of GSL pathway genes

**Figure 3:**
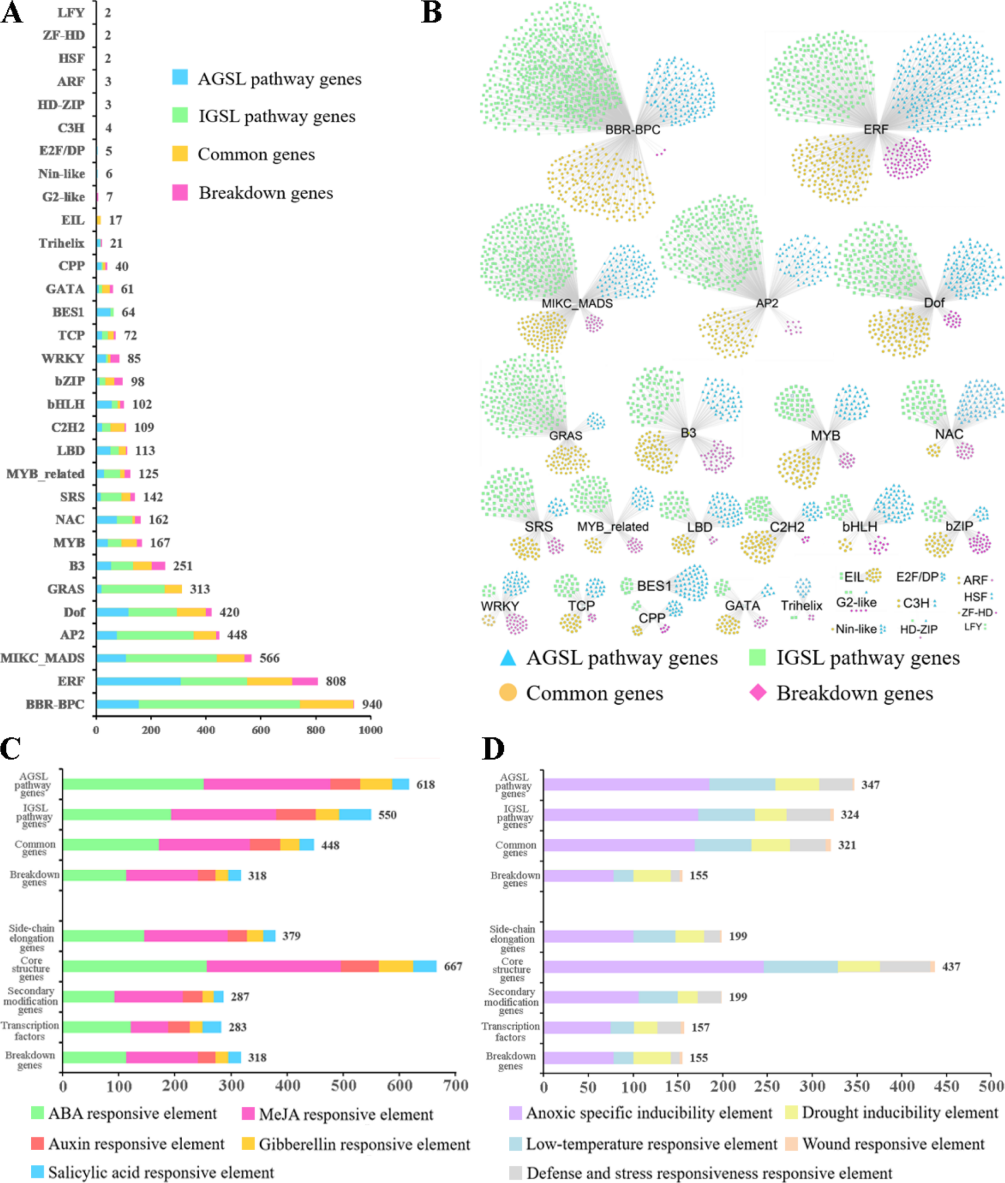
*Cis*-acting regulatory elements (CREs) and transcription factor (TF) binding site analysis in promoters of GSL pathway genes. **(A)** The TF gene families with potential binding sites in the promoter regions of GSL pathway genes. **(B)** The distribution of TF gene families with potential sites in the promoter regions of the four processes of GSL pathway. **(C)** The *cis*-elements related to hormone responsiveness in the promoter regions of GSL pathway genes. **(D)** The *cis*-elements related to stress responses in the promoter regions of GSL pathway genes. The X-axis represents the gene number.

Up to 5158 TFs from 31 families were predicted to be involved in the regulation of GSL biosynthesis genes (**Figure 3**; **Supplementary Table S5**), with 1314 TFs in the AGSL pathway, 2337 TFs in the IGSL pathway, 799 TFs in the co-substrate pathway, and 403 TFs in breakdown pathway. Among them, BARLEY B-RECOMBINANT/BASIC PENTACYSTEINE (BBR-BPCs), Ethylene Responsive Factor (ERF), MIKC-type MCM1-AGAMOUS-DEFICIENSSRF-box (MIKC_MADS), APETALA2 (AP2), DNA binding with one finger (Dof) and B3 families had a relatively large number of potential target genes which were mainly involved in AGSL and IGSL pathways (**Figure 3A**). BBR-BPCs (940 genes) were the most abundant TFs, whose targets were mainly involved in all GSL pathways; ERF family members (808 genes) primarily regulated the genes involved in core structure biosynthesis processes in both AGSL and IGSL pathways; MIKC-MADS family members (566 genes) mainly participated in the regulation of AGSL and IGSL core structure biosynthesis, along with secondary modification of AGSL (**Supplementary Table S5**); AP2 family members (448 genes) have potential target sites in the genes in side-chain elongation of AGSL; while the targets of GRAS family members (313 genes) only in sulfate assimilatory process of AGSL and IGSL core structure biosynthesis (**Supplementary Table S5**). Other types of TFs, such as members of MYB, bHLH, and bZIP families may play potential regulatory roles in the GSL pathway. Moreover, the genes in the IGSL pathway generally have a relatively larger number of potential upstream TFs than those in the AGSL pathway (**Figure 3B**), exhibiting a more complex regulatory network.

Based on the PlantCARE database, a total of 69 types of 26644 *cis*-acting regulatory elements (CREs) were identified in the promoter regions (-1500bp) of the GSL biosynthesis genes (**Supplementary Table S6**). The majority of them were promoter core regulation elements, such as CAAT-box and GC-box, and light-responsive elements. Meanwhile, a mass of hormone responsive *cis*-elements were obtained (**Figure 3C**; **Supplementary Table S6**), including 730 abscisic acid (ABA) responsive elements (ABRE), 703 methyl jasmonate (MeJA) responsive elements (CGTCA-motif, GACG-motif), 208 auxin responsive elements (AuxRE, AuxRR-core, TGA-box and TGA-element), 156 gibberellin responsive elements (GARE-motif, TATC-box and P-box), and 137 salicylic acid responsive elements (TCA-element, SARE). This suggested the important roles of hormones in regulating the biosynthesis of GSLs in *B. napus*. In addition, 1147 *cis*-elements related to adverse stress responses were identified as well (**Figure 3D**; **Supplementary Table S6**). For example, 587 antioxidant response elements (AREs) involved in anaerobic induction, 222 LTRs in low-temperature responsive, 169 MYB binding sites involved in drought-inducibility (MBSs) in drought-inducibility, and 136 TC-rich repeats in defense and stress responsive, were identified in our results. Conversely, a few *cis*-elements involved in the regulation of plant growth and development were identified, such as GCN4**_**motif (involved in endosperm expression), CAT-box (related to meristem expression), and HD-ZIP1 (involved in differentiation of the palisade mesophyll cells).

### 3.4 Comparative transcriptome atlas between ZS11 and ZY821 at different developmental stage

**Figure 4:**
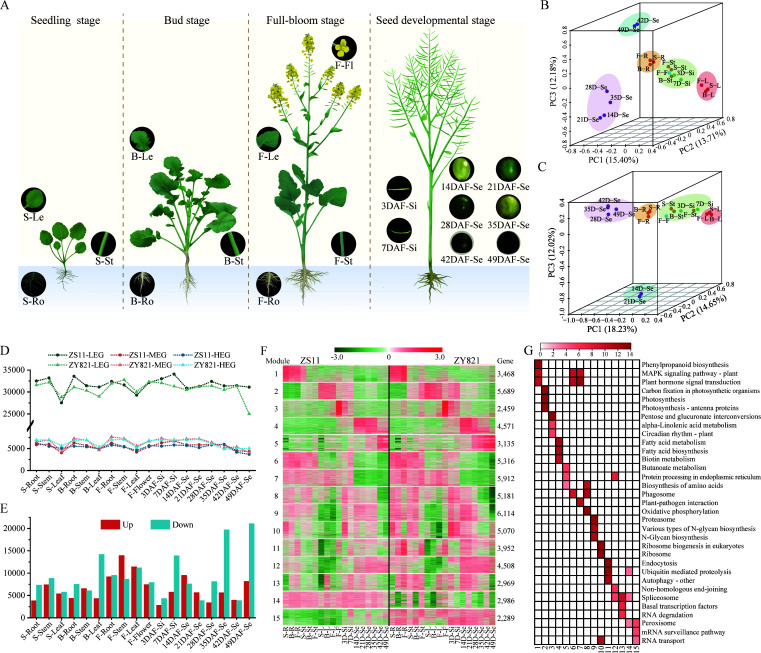
Transcriptome analysis of Zhongshuang 11 (ZS11) and Zhongyou 821 (ZY821) at different developmental stages. **(A)** The samples of rapeseed Zhongshuang11 (ZS11) and Zhongyou 821 (ZY821) varieties used in spatio-temporal transcriptome. These samples include root (Ro), leaf (Le), stem (St), flower (Fl), silique (Si), and seed (Se), which harvested from seedling (S), bud (B), full-bloom (F), and seed developmental stages covering the entire life cycle of ZS11 and ZY821 respectively. DAF, days after flowering. **(B)** and **(C)** Principal component analysis (PCA) based on the expression profile of ZS11 and ZY821 respectively. **(D)** Gene expression grading for each sample of ZS11 and ZY821 varieties. LEG: low expression gene (FPKM<1), MEG: middle expression gene(1<fpkm10). The Y-axis represents the gene number. **(E)** Differentially expressed gene (DEG) of ZS11 and ZY821 (up indicates the expression in ZY821 is higher than in ZS11; down represent the expression in ZS11 is higher than in ZY821). **(F)** The k means clustering analysis of DEGs. **(G)** The KEGG pathway enrichment of 15 co-expression modules.

We constructed a high-quality RNA-seq dataset of roots, stems, leaves, flowers, silique (3d, 7d) and seeds (14, 21, 28, 35, 44 and 49d) of ZY821 and ZS11 at different developmental periods (seed, bud, flower and seed maturation) (**Supplementary Table S7**; **Figure 4A**). In all, 124,629 and 112,675 transcripts were obtained in ZS11 and ZY821, respectively, including 102,566 overlapped transcripts and 22,063 and 10,109 ZS11- or ZY821-specific transcripts. In ZS11, we detected a total of 88,740 expressed transcripts, including 41,893 and 46,847 transcripts distributed on the A_n_ and C_n_- subgenomes, respectively (303 transcripts scattered on unanchored scaffolds). The number of transcripts detected in the 18 samples ranged from 55,893 to 68,492. In ZY821, a total of 79,341 transcripts were identified, with 37,422 and 41,589 transcripts distributed on the A_n_ and C_n_ subgenomes, respectively, while 330 transcripts scattered on unanchored scaffolds. The number of transcripts varied from 42,952 to 58,394 across 18 samples.

Principal component analysis (PCA) clustered the samples from both ZS11 (**Figure 4B**) and ZY821 (**Figure 4C**) into five highly similar groups (G1-G5), respectively, corresponding to different tissues/organs. The most significant differences were observed in the developmental seed gradient, where the genes in 28 DAF-Se and 35 DAF-Se showed delay expression changes in ZY821 (**Figures 4B, C**), suggesting the most transcriptional changes occurred in seeds around 28-35 DAF. To gain further insights into the dynamic activity of the global transcriptome between ZS11 and ZY821, we clustered the 63,594 shared-expressed genes into 15 co-expression modules using the k means clustering algorithm (**Figure 4F**). Genes belonging to module 1-13 (91.76%) showed a similar expression pattern between cultivars, while those in module 14 and 15 had entirely different expression patterns between ZS11 and ZY821, proving that most genes had conserved expression patterns during the domestication. The corresponding functional categories reflected the biological processes of each module and were characterized by organ(s) (**Figure 4G**). Genes in modules 1-5 were preferentially expressed in one organ and represented the particular functions for the corresponding organs, for instance, the genes in module 2 with dominant expression in leaves were dominated by photosynthetic category; while those in modules 4 and 5 having predominant transcriptional activity in early (14-35 DAF) and late (42-49 DAF) developmental stage of seeds were dominated by fatty acid and proteins and amino acid related categories, respectively (**Figure 4G**), revealing the altered features between two cultivars during the domestication.

To further unravel the transcriptional activity and functional transition difference along development between ZS11 and ZY821, we detected DEGs in each of the 18 tissues/organs. Totally, 56,599 non-redundant DEGs were obtained, the number of which exhibited strong spatial-temporal characteristics (**Figure 4D**). The largest number of DEGs was detected at the seed developmental stage while the lowest was observed at the seedling stage (**Figure 4E**). For a given development stage, the DEGs exhibited a strong organ-specificity. For example, 69.28% DEGs were root- or stem-specific at the seedling stage. Most DEGs were obtained from seed samples, whereas the fewest were from roots. For a given organ, the DEGs have less overlap among the samples, showing a developmental specificity, with the number generally increased gradually along with the development (**Figure 4E**). Functional annotation analysis showed that the DEGs in each sample were co-substrate enriched in specific biological functions associated with organ(s), while generally differed across the developmental stages for the same organ (**Figure 4G**), showing a dynamic functional transition feature along the development process. Moreover, a larger number of DEGs were presented in 12 of the 18 samples in ZS11 (**Figure 4E**), suggesting a predominant advantage in gene expression level in ZS11 than ZY821. Notably, the number of DEGs fluctuated dramatically at different seed developmental stages (**Figure 4E**). This is most likely attributed to seeds being the predominant selection trait in rapeseed breeding history as an oil seed crop.

Overall, we constructed a high-quality transcriptome dataset related to development in ZS11 and ZY821. The differences between the two cultivars may be attributed to the superposition of diverse expression patterns in each organ at different stages, especially in seeds.

### 3.5 Comparative expression analysis of GSL biosynthesis pathway between ZS11 and ZY821

Based on the above transcriptome dataset constructed in this study, we compared the expression profiles of GSL biosynthesis genes in different organs between ZY821 and ZS11 cultivars. The results showed that the GSL biosynthesis genes were highly expressed in vegetative organs, such as roots, stems, and leaves, while lowly expressed in flowers and seeds, indicating GSLs were biosynthesized in both below- and above-ground vegetative tissues (**Supplementary Figure S1**).

**Figure 5:**
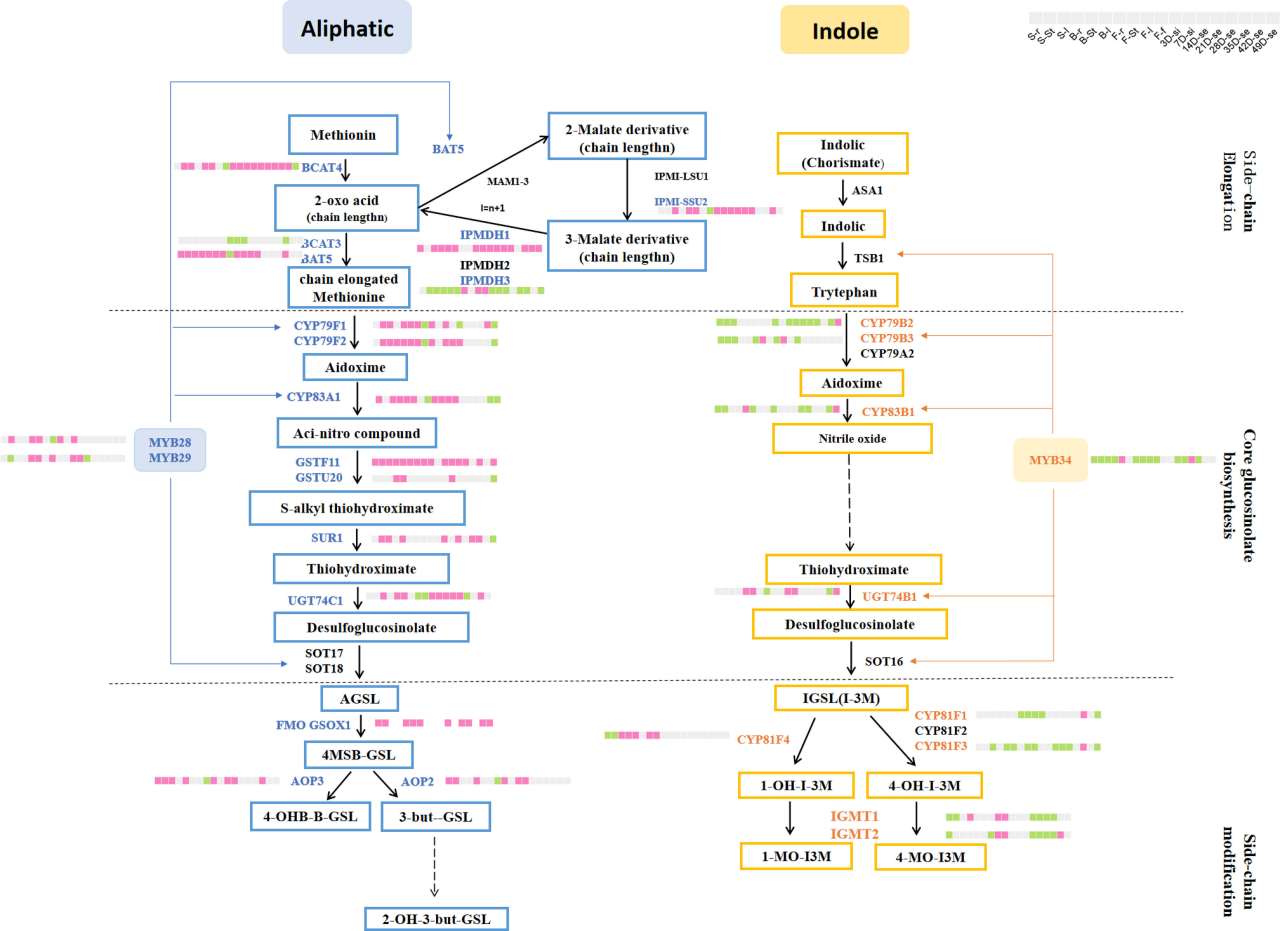
The DEGs between ZY821 and ZS11 in the AGSL and IGSL pathways. Pink box represents the DEGs have higher expression levels in ZY821 than in ZS11; Green box represents the expression levels of the DEGs in ZS11 higher than in ZY821; Gray box represents these with similar expression value in ZS11 and ZY821.

A total of 188 GSL genes were differentially expressed in more than one organ between ZS11 and ZY821 (**Supplementary Table S8**), including 162 structural genes (such as *BnaBACT4*, *BnaCYP79F1*, and *BnaCYPB3A1*) and 26 TFs (such as *BnaDof1*, *BnaMYC4*, and *BnaMYB28*). Among them, there were 57 genes in the AGSL pathway, 52 genes in the IGSL pathway, 30 genes in the co-substrate pathway, and 31 genes in the breakdown pathway. In each organ, the DEGs in the AGSL biosynthesis pathway were mainly up-regulated and their corresponding expression values in ZY821 were commonly higher than those in ZS11. This trend is consistent with the fact that ZY821 has a high GSL content (Lu et al., 2019). For example, the homolog of *Arabidopsis* AGSL regulating key gene (*MYB28*), *BnaMYB28-1-A-AG*, exhibited higher expression levels in five samples of ZY821 rather than ZS11 (one sample), and the homologs of *Arabidopsis CYP79F1* and *CYP83A1* (*BnCYP79F1-1-C-AG, BnCYP83A1-1-A-AG, BnCYP83A1-2-A-AG, BnCYP83A1-1-C-AG* and *BnCYP83A1-2-C-AG*) that were directly activated by MYB28 were mainly highly expressed in ZY821 rather than ZS11 as well (**Figure 5**). It was proved that the overexpression lines of *BnaMYB28* showed an increased GSL content while the *bnamyb28* mutant exhibited a decreased GSL content in *B. napus* (Zhou et al., 2023), further supporting the confidence of our results. On the contrary, the expressions of the DEGs in the IGSL biosynthesis pathway were generally down-regulated in most organs in ZY821 than in ZS11 (**Figure 5**) (Lu et al., 2019). For example, the DEGs of the *BnaMYB34* gene whose homolog in *Arabidopsis* is regarded as the key TF for IGSL (Sawada et al., 2009), exhibited lower expression in ZY821 than in ZS11 in most organs investigated (**Figure 5**). Accordingly, the homologs of *Arabidopsis CYP79B2* and *CYP83B1* (*BnCYP79B2-2-C-IG, BnCYP79B2-3-C-IG, BnCYP83B1-1-A-IG* and *BnCYP83B1-1-C-IG*) that were directly activated by MYB34 were mainly lowly expressed in ZY821 than in ZS11 (**Figure 5**). The main type of GSL content in *B. napus* was AGSL, accounting for more than 90% (Tang et al., 2023). Therefore, the higher expression of AGSL genes in ZY821 resulted in the high GSL content. The IGSL content was slightly lower in ZS11 compared to ZY821 (Tang et al., 2023). However, the higher expression pattern of GSL biosynthesis genes was found in ZS11 than in ZY821. This result indicated that the difference in IGSL content between ZS11 and ZY821 is not in the synthesis step, but may be in the transport or degradation step.

To further identify the key genes that lead to the difference in GSL content between ZY821 and ZS11, we obtained 65 DEGs that showed significant regulation directory (the number of up-regulated organs/down-regulated > 2, up; the number of up-regulated organs/down-regulated < 0.5, down) (**Supplementary Table S9**). Most of them belong to the core structure pathway and exhibited a higher expression in ZY821 than in ZS11, such as *BnCYP79F1-1-C-AG*, *BnCYP79F2-1-A-AG*, *BnCYP83A1-1-A-AG, BnCYP83A1-2-A-AG, BnCYP83A1-1-C-AG* and *BnCYP83A1-2-C-AG* in AGSL core structure pathway were up-regulated in almost all organs of ZY821, while *BnCYP79B2-2-C-IG, BnCYP79B2-3-C-IG, BnCYP79B3-1-A-IG,BnCYP83B1-1-A-IG* and *BnCYP83B1-1-C-IG* in IGSL core structure pathway were down-regulated in most of the organ in ZY821 (**Figure 5**), indicating their important roles in GSLs biosynthesis. Consistent with our speculation, some candidate genes had already been confirmed to play a key role in regulating GSL biosynthesis in *B. napus*, e.g., the homologs of *CYP79B2*, *CYP83A1*, and *GSTF11* genes (*BnCYP79B2-2-C-IG, BnCYP79B2-3-C-IG, BnCYP83A1-1-A-AG, BnCYP83A1-2-A-AG, BnCYP83A1-1-C-AG BnCYP83A1-2-C-AG* BnGSTF11-1-A-AG and BnGSTF11-1-C-AG) (Mikkelsen et al., 2000; Naur et al., 2003; Reintanz et al., 2001; Zang et al., 2008; Zhang et al., 2021), supporting the reliability of our results.

## 4 Discussion

Darwin’s theory of evolution suggested that all plants evolved from a common ancestor, having a basic biological function. During the long-term evolution process, plants gradually diversified and formed a great plant kingdom through a series of novelties, including new traits, new genetic variation, etc, resulting in the complex global biological system, which attributed to the lens of genome-wide duplication, gene duplication, and loss, gene families or pathway differentially expansion. As ubiquitous compounds in the plant kingdom, plant secondary metabolites also play an important role in plant major novelties and specific features, by increasing the adaptive evolution of terrestrial systems and playing myriad ecological roles in defense against herbivores, pathogens etc. For example, isoflavonoids are the specific secondary metabolites distributed in leguminous plants; solanine are only product from Solanaceae plants (Hassan et al., 2021). The GSLs were demonstrated to be distributed merely in Brassicaceae with important ecological roles in herbivores and pathogens resistance, etc (Kliebenstein et al., 2001). Similarly, the species-specificity was also found in the gene family level, such as the subfamilies of S74-S78 in the R2R3-MYB gene family were specifically identified in Brassicaceae (Li et al., 2020). However, this hypothesis remains to be confirmed at a global level focusing on the whole GSL pathway yet, including the structural genes and regulatory genes in major representative plants, as our current understanding is only derived from scattered experimental studies of gene function and/or GSL contents in some plant species, especially in model plant *Arabidopsis*. In the present study, aimed to gain insights into the evolutionary history of this pathway in plants, we conducted a genome-wide identification of the GSL pathway genes in plant genomes in Phytozome. As expected, the homologs of this pathway genes were only obtained in Brassicaceae species. Thus, we focus on the results in 14 representatives, including 13 dicot species (nine Brassicaceae and four closely related non-Brassicaceae species) and one monocot species. In all, 1280 GSL pathway genes were obtained, however, the majority of which only existed in the Brassicaceae species (**Figure 1**). The gene distribution trend of this pathway is consistent with the fact that GSL were only detected in Brassicaceae species to date (Kliebenstein et al., 2001), and the diversification of GSL biosynthesis genes are closely tied to the evolutionary history of Brassicaceae. These results demonstrated the lineage-specific origin of the GSL pathway and distribution of GSLs in plant kingdom, which can serve as a classic example of adaptive evolution in plants.

As a group of important plant secondary metabolites that mainly exist in the Brassicaceae family, GSLs and their degradation products are recognized to have a wide range of biological activities, such as resistance against pathogens, insects, fungi, and herbivores. However, their degradations are considered to be detrimental to human health, hence the breeding of low-GSL-content *B. napus* variety is an important goal in *B. napus*. Accordingly, many *B. napus* cultivars with low-GSL content have been developed by traditional breeding methods over the past three decades worldwide, such as ZS11 (Kittipol et al., 2019). However, this increases the risk of pests, diseases, and bird damage occurrence rate in *B. napus* production. Undoubtedly, it is more efficient to regulate GSL biosynthesis and accumulation in a given spatio-temporal manner of *B. napus* through modulating key genes by molecular breeding strategies. For instance, it was demonstrated that knocking out key genes in the GSL biosynthesis pathway (e.g., *BnaMYB28* and *BnaCYP79F1*) could reduce GSL contents in *B. napus* by CRISPR/Cas9 (Cong et al., 2013). Moreover, it is well-known that GSL content in seeds is significantly higher than those in other tissues/organs. In *B. napus*, the GSL content in seeds was about 180 μmol/g, whereas the content in leaves was only 10-20 μmol/g (Petersen et al., 2002). Seeds synthesize very limited GSLs. The majority of GSLs in *B. napus* seeds are transported from vegetative organs, e.g., leaves, stems, or siliques (Nour-Eldin et al., 2012). Accordingly, some studies have successfully modulated the transport of GSLs in plants to address the purpose of engineering GSLs. For instance, modulating three genes that were involved in GSL transport, *BocGRT1a*, *BocGRT1b*, and *BocGRT1c*, could control the GSL transport processes in *B. oleracea* (Jiang et al., 2019). Six homologs of *GRT1* (*BnaGRT1.1-1.6*) and seven homologs of *GRT2* (*BnaGRT2.1-2.7*) were identified in *B. napus*, and only *BnaGRT1.2* and *BnaGRT1.6* showed a higher expression in ZY821 in only one sample. These results indicated that the different GSL content mainly resulted in the biosynthesis pathway rather than the transport process. Nevertheless, the identification of key genes involved in the GSL pathway is an important goal for *B. napus* molecular breeding.

RNA sequencing is regarded as the most useful method to screen gene resources at the transcriptome level, nowadays. For example, the homologs of *Arabidopsis MYB28*, *MYB34*, and *AOP3*, which are involved in controlling seed GSL contents, were proven to be the common and ecotype-specific genes for seed GSL contents in *B. napus* by RNA-seq method (Wei et al., 2019). By the same method, we obtained 65 DEGs from the 344 *B. napus* GSL pathway genes that may be the potential key genes in regulating high and low GSL contents based on the transcriptome atlas of the high- (ZY821) and the low-GSL-content (ZS11) cultivars constructed in this study. Some of these candidates had been functionally confirmed, which further supported our results. For instance, the *CYP79F1* gene was proved to involve in long and short-chain AGSL biosynthesis whose mutant showed decreased GSL content in *Arabidopsis* (Wang et al., 2020); a double mutation of *gstu20* and *gstf11* leads to a loss of AGSLs in *Arabidopsis* (Zhang et al., 2021). Many TFs were also proved to have important roles in GSL biosynthesis, especially *R2R3-MYB* genes, by directly regulating expressions of the structural genes in the GSL pathway, such as *Arabidopsis MYB28*, *MYB29*, and *MYB67* in the S12 subfamily of the R2R3-MYB gene family (Li et al., 2013; Sonderby et al., 2010). Among these, *MYB28* encoded the positive regulator for the transcription of *CYP79F1/F2*, *CYP83A1*, and *BAT5* genes in the AGSL biosynthesis pathway (Gigolashvili et al., 2009). Meanwhile, *MYB34*, *MYB51*, and *MYB122* genes in the same subfamily are responsible for IGSL biosynthesis, with *MYB34* acting as the main regulator gene (Sawada et al., 2009; Frerigmann et al., 2014). Our previous studies have demonstrated that MYB gene families have undergone constant large expansion along with the increasing complexity of angiosperms, resulting in many lineage- or species-specific subfamilies (Du et al., 2015). The newly origin subfamilies have evolved specific roles in diverse processes, such as abiotic stresses, secondary metabolism, etc (Du et al., 2015). Even different clades in the same subfamily underwent functional diversification during the evolution (Du et al., 2015; Li et al., 2020). For example, as mentioned above, the two clades of the S12 subfamily represent the MYB28 homologs in regulating AGSL biosynthesis and the MYB34 homologs in regulating IGSL biosynthesis. Based on our RNA-seq data, we found that the *BnaMYB28* gene and the AGSL-related structural genes (e.g., *CYP79F1/F2* and *CYP83A1*) were highly expressed in ZY821 than in ZS11, whereas *BnaMYB34* and the IGSL related structural genes (e.g., *CYP79B2/B3* and *CYP83B1*) were highly expressed in ZS11 than ZY821 (**Figure 5**), indicating the important roles of them in GSLs biosynthesis in *B. napus*. Together, the genes identified in this study represent a potentially valuable gene source for future gene functional dissection and molecular breeding programs of GSLs content in *B. napus*.

## 5 Conclusion

In this study, we investigated the distribution of GSL pathway genes in main land plants with available sequenced genome data and demonstrated the majority of genes merely existed in Brassicaceae. The WGD event was the main driving force for gene amplification of the GSL pathway in *B. napus*. The genes in the GSL pathway in *B. napus* may be regulated by various TFs and *cis*-elements and were mainly responsive to abiotic stress and hormonal induction. The genes involved in the AGSL pathway might be the main factors leading to the differences in GSL content between ZY821 and ZS11, by comparing the RNA-seq dataset of the two cultivars at different developmental stages. Sixty-five significant DEGs may contribute to the difference in GSL content between the two high- and low-GSL content cultivars.

## Data Availability

The data presented in the study are deposited in the BioProject of NCBI Search database repository, accession number PRJNA612898.
