# Supplementary material for: Evolution and comparative transcriptome analysis of glucosinolate pathway genes in Brassica napus L
Source: Front Plant Sci. 2024 Dec 10;15:1483635. doi: 10.3389/fpls.2024.1483635 (PMC11666375; doi:10.3389/fpls.2024.1483635)
Supplement: Supplementary file 1 [file DataSheet1.pdf]

Supplementary Material

1 Supplementary Figures and Tables

1.1 Supplementary Figures

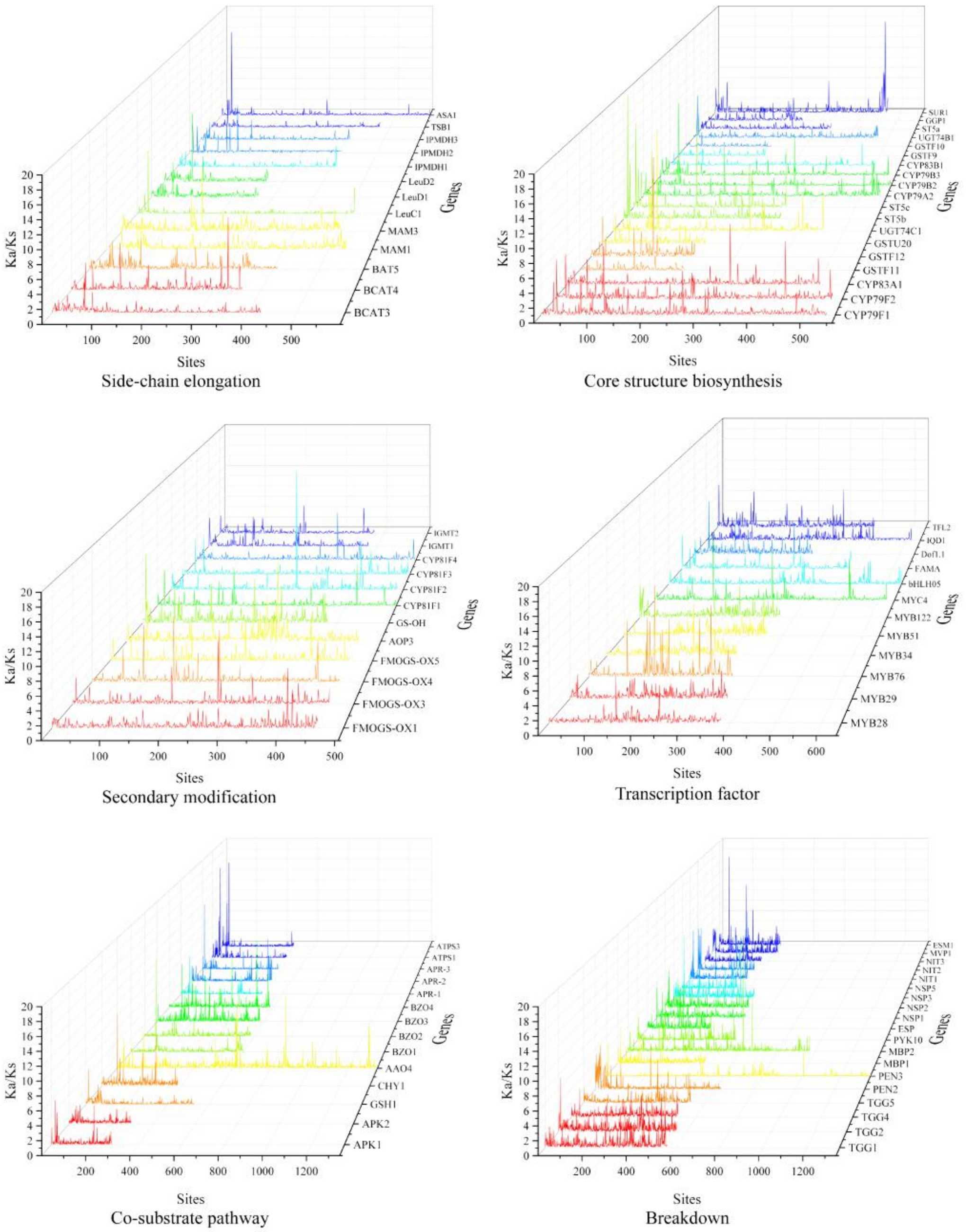

Supplementary Figure 1.. Selection pressure analyses of GSL pathway genes.

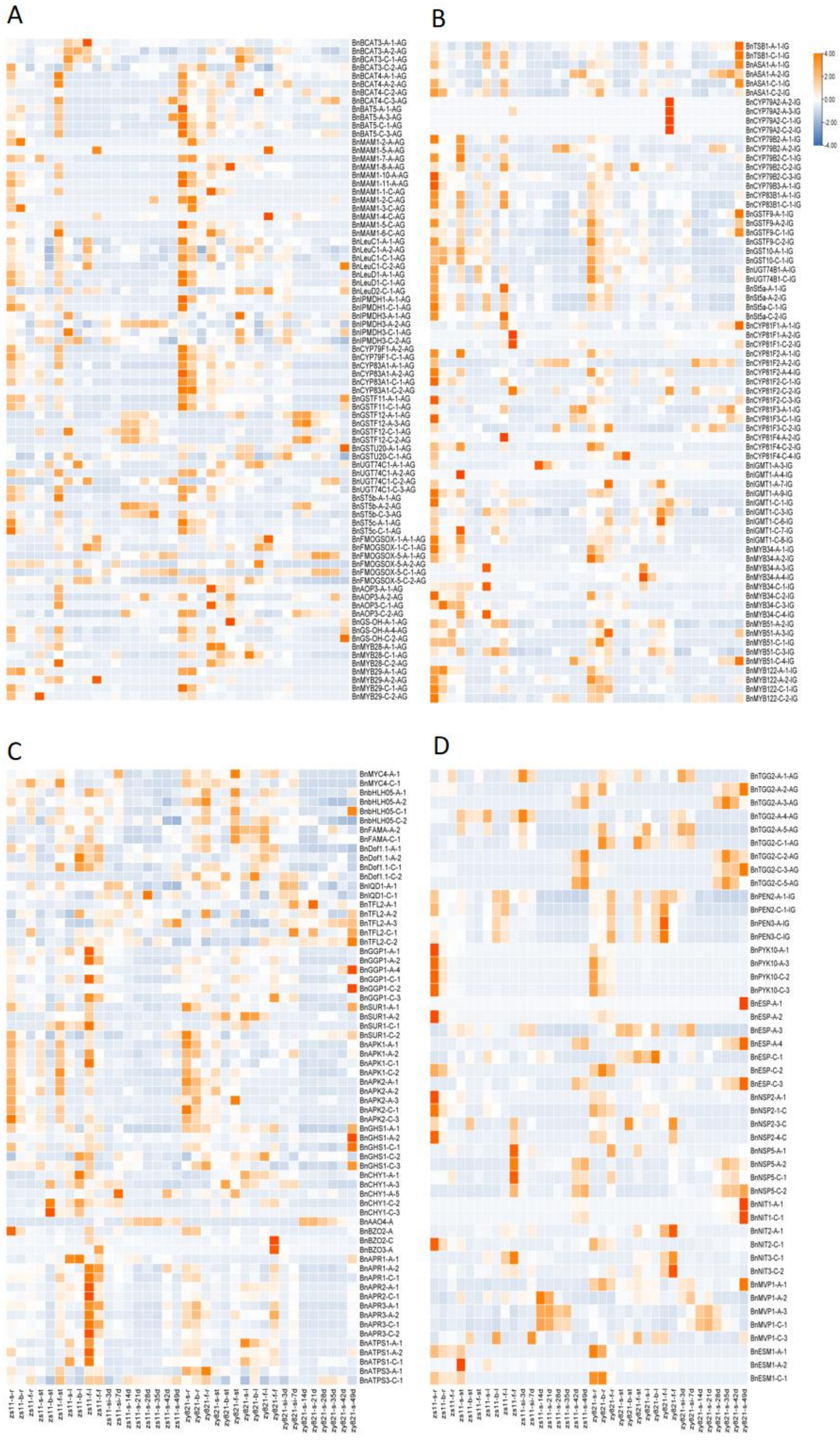
A
B
C
D

Supplementary Figure 2. Expression profiles of GSL biosynthetic genes at different developmental stages.

1.2 Supplementary Tables

Supplemental Table S1. Glucosinolate (GSL) biosynthetic genes in 14 land plants.

Supplemental Table S2. Selective pressure of GSL pathway genes in 14 land plants.

Supplemental Table S3. The 344 GSL biosynthetic genes from Brassica napus identified in this study.

Supplemental Table S4. Syntenic relationship of GSL biosynthesis genes in B. napus, Brassica rape, and Brassica oleracea.

Supplemental Table S5. Regulatory network of the 344 GSL biosynthetic genes.

Supplemental Table S6. Cis-acting regulatory elements of the 344 GSL biosynthetic genes.

Supplemental Table S7. Quality control information of Zhongyou 821 (ZY821) and Zhongshuang11 (ZS11) RNA-seq dataset in *B. napus*.

Supplemental Table S8. The 188 GSL genes were differentially expressed in more than one organ between ZS11 and ZY821.

Supplemental Table S9. The 65 candidate genes that may mainly contribute to GSL content difference in ZY821 and ZS11.
